# Crystal structure of *catena*-poly[[di­aqua­bis­(4-formyl­benzoato-κ*O* ^1^)cobalt(II)]-μ-pyrazine-κ^2^ *N*:*N*′]

**DOI:** 10.1107/S205698901500403X

**Published:** 2015-03-04

**Authors:** Gülçin Şefiye Aşkın, Fatih Çelik, Nefise Dilek, Hacali Necefoğlu, Tuncer Hökelek

**Affiliations:** a Department of Physics, Hacettepe University, 06800 Beytepe, Ankara, Turkey; b Department of Chemistry, Kafkas University, 36100 Kars, Turkey; c Aksaray University, Department of Physics, 68100, Aksaray, Turkey

**Keywords:** crystal structure, cobalt(II), transition metal complexes, benzoic acid derivatives, one-dimensional coordination polymer

## Abstract

In the title polymeric compound, [Co(C_8_H_5_O_3_)_2_(C_4_H_4_N_2_)(H_2_O)_2_]_*n*_, the Co^II^ atom is located on a twofold rotation axis and has a slightly distorted octa­hedral coordination sphere. In the equatorial plane, it is coordinated by two carboxyl­ate O atoms of two symmetry-related monodentate formyl­benzoate anions and by two N atoms of two bridging pyrazine ligands. The latter are bis­ected by the twofold rotation axis. The axial positions are occupied by two O atoms of the coordinating water mol­ecules. In the formyl­benzoate anion, the carboxyl­ate group is twisted away from the attached benzene ring by 7.50 (8)°, while the benzene and pyrazine rings are oriented at a dihedral angle of 64.90 (4)°. The pyrazine ligands bridge the Co^II^ cations, forming linear chains running along the *b*-axis direction. Strong intra­molecular O—H⋯O hydrogen bonds link the water mol­ecules to the carboxyl­ate O atoms. In the crystal, weak O—H_water_⋯O_water_ hydrogen bonds link adjacent chains into layers parallel to the *bc* plane. The layers are linked *via* C—H_pyrazine_⋯O_form­yl_ hydrogen bonds, forming a three-dimensional network. There are also weak C—H⋯π inter­actions present.

## Abstract 2

The Co^II^ atom in this one-dimensional coordination polymer, with aqua, 4-formyl­benzoate and bridging pyrazine ligands, is located on a twofold rotation axis and has a slightly distorted octa­hedral coordination sphere. Strong intra­molecular O—H⋯O hydrogen bonds link the water mol­ecules to the carboxyl­ate O atoms.

## Chemical context   

The structural functions and coordination relationships of the aryl­carboxyl­ate ion in transition metal complexes of benzoic acid derivatives change depending on the nature and position of the substituent groups on the benzene ring, the nature of the additional ligand mol­ecule or solvent, and the medium of the synthesis (Adiwidjaja *et al.*, 1978 ▸; Antsyshkina *et al.*, 1980 ▸; Nadzhafov *et al.*, 1981 ▸; Shnulin *et al.*, 1981 ▸). Transition metal complexes with biochemically active ligands frequently show inter­esting physical and/or chemical properties and, as a result, they may find applications in biological systems (Antolini *et al.*, 1982 ▸). Some benzoic acid derivatives, such as 4-amino­benzoic acid, have been extensively reported in coordination chemistry, as bifunctional organic ligands, due to the varieties of their coordination modes (Chen & Chen, 2002 ▸; Amiraslanov *et al.*, 1979 ▸; Hauptmann *et al.*, 2000 ▸).

In this context, we report the synthesis and crystal structure of the title compound, [Co(C_8_H_5_O_3_)_2_(C_4_H_4_N_2_)(H_2_O)_2_]_*n*_, which is isotypic with its Cu^II^ (Çelik *et al.*, 2014*a* ▸) and Ni^II^ (Çelik *et al.*, 2014*b* ▸) analogues.

## Structural commentary   

**Figure 1:**
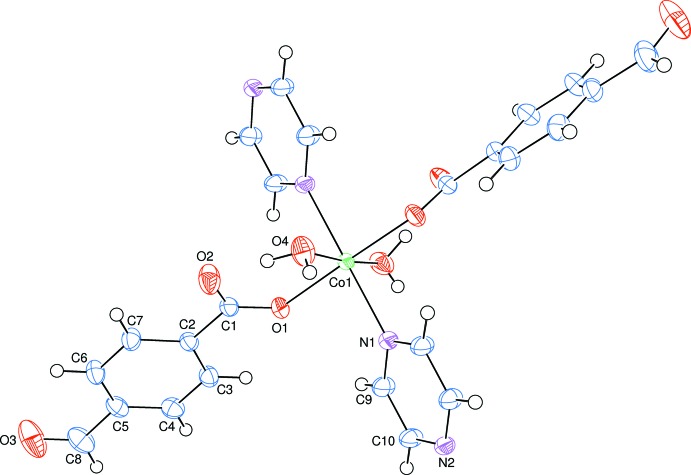
A view of the coordination environment around the Co^II^ atom of the title mol­ecule, showing the atom labelling. Displacement ellipsoids are drawn at the 50% probability level. The twofold rotation axis bis­ects atoms Co1, N1 and N2. Non-labelled atoms are generated by the symmetry code −*x* + 2, *y*, −*z* + 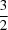.

**Figure 2:**
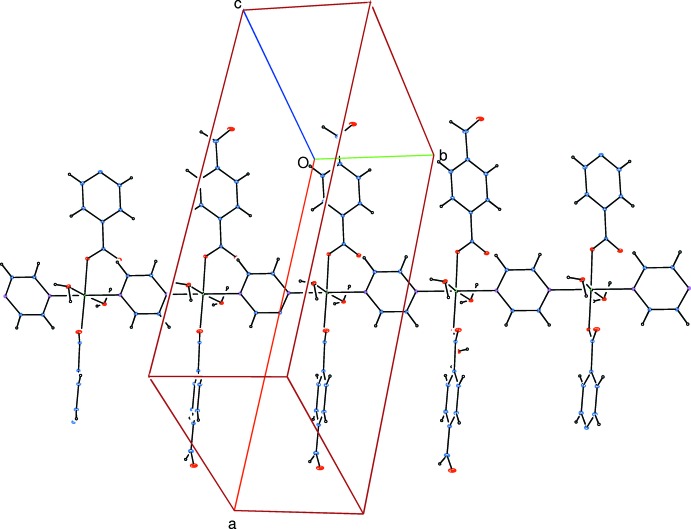
A partial view of the crystal packing of the title compound.

The asymmetric unit of the title compound contains a Co^II^ ion, one formyl­benzoate (FB) anion, one water mol­ecule and half of a pyrazine mol­ecule. Atoms N1 and N2 of the pyrazine ligand and Co1 are located on a twofold rotation axis (Fig. 1 ▸). The pyrazine ligands bridge adjacent Co^II^ ions, forming polymeric chains running along the *b-*axis direction (Fig. 2 ▸). The distance between symmetry-related Co^II^ ions [Co1⋯Co1^iii^; symmetry code: (iii) *x*, *y* + 1, *z*] is 7.1193 (4) Å.
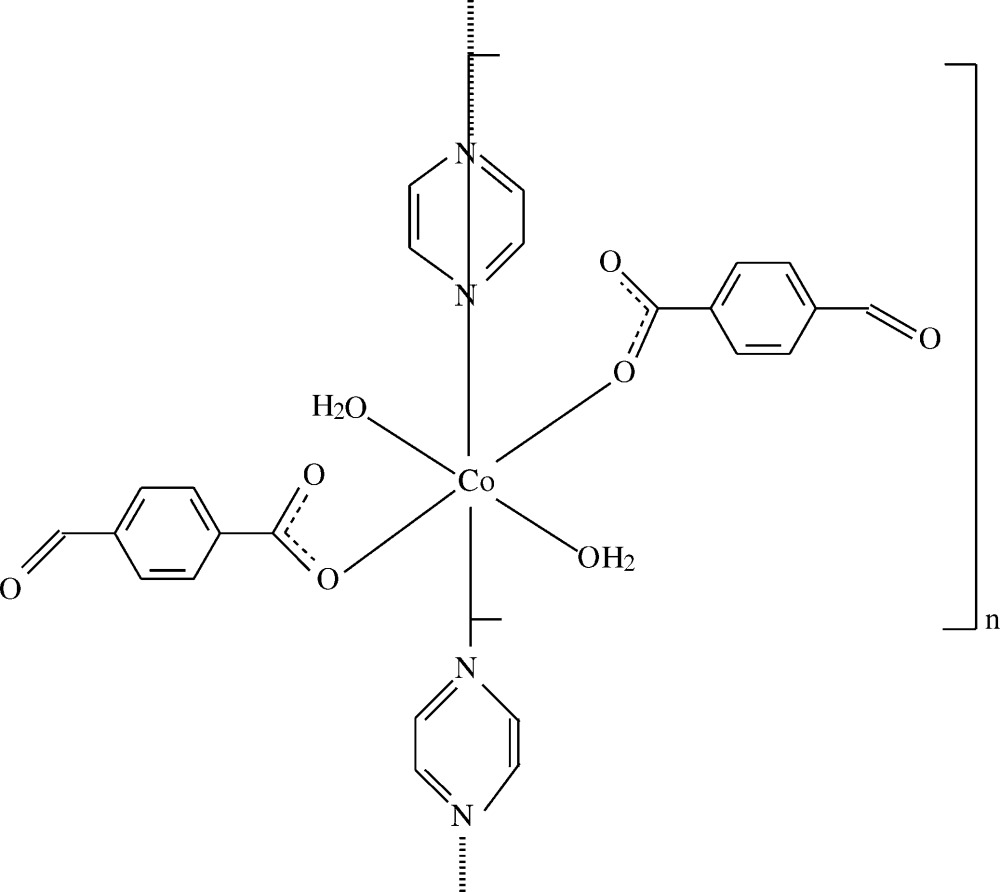


The equatorial plane of the Co^II^O_4_N_2_ coordination sphere is composed of two carboxyl­ate O atoms [O1 and O1^i^; symmetry code: (i) 2 − *x*, *y*, 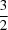 − *z*] of two symmetry-related monodentate formyl­benzoate anions and two N atoms [N1 and N2^ii^; symmetry code: (ii) *x*, −1 + *y*, *z*] of two bridging pyrazine ligands, which are bis­ected by the twofold rotation axis. The axial positions are occupied by two O atoms (O4 and O4^i^) of the coordinating water mol­ecules.

The near equality of the C1—O1 [1.272 (2) Å] and C1—O2 [1.245 (2) Å] bonds in the carboxyl­ate group indicates a delocalized bonding arrangement, rather than localized single and double bonds. The Co—N bond length is 2.165 (9) Å, while the Co—O bond lengths are 2.0551 (9) Å (for benzoate oxygen) and 2.1491 (11) Å (for water oxygen), close to standard values. The Co1 atom is displaced by 0.1034 (2) Å from the mean plane of the carboxyl­ate group (O1/C1/O2). The dihedral angle between the carboxyl­ate group and the adjacent benzene ring *A* (C2–C7) is 7.50 (8)°, while the benzene and pyrazine rings are oriented at a dihedral angle of 64.90 (4)°.

## Supra­molecular features   

**Figure 3:**
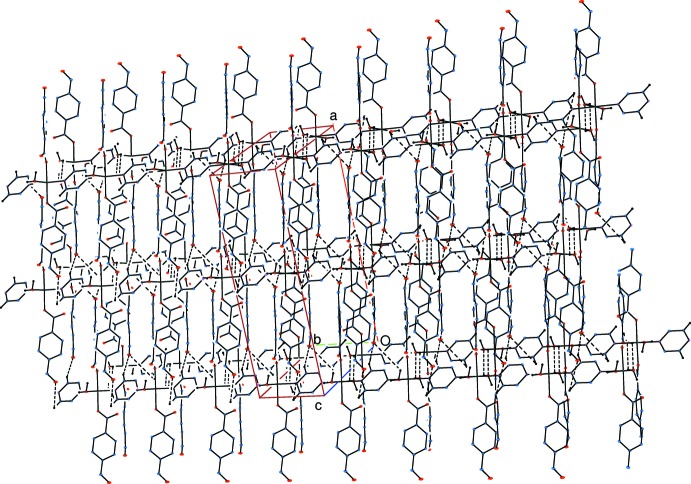
Part of the crystal structure. Inter­molecular hydrogen bonds are shown as dashed lines. Non-bonding H atoms have been omitted for clarity.

Strong intra­molecular O—H⋯O hydrogen bonds (Table 1 ▸) link the water mol­ecules to the non-coordinating carboxyl­ate oxygen atoms. In the crystal, weak O—H_water_⋯O_water_ hydrogen bonds (Table 1 ▸) link adjacent chains into layers parallel to the *bc* plane. The layers are linked *via* C—H_pyrazine_⋯O_form­yl_ hydrogen bonds, forming a three-dimensional network (Fig. 3 ▸). There are also weak C—H⋯π inter­actions present (Table 1 ▸).

## Refinement   

The experimental details including the crystal data, data collection and refinement are summarized in Table 2 ▸. Atoms H41 and H42 (for H_2_O) were located in a difference Fourier map and were refined freely. The methine H atom was also located in a difference Fourier map and the C—H distance restrained to 0.984 (13) Å. The aromatic C-bound H atoms were positioned geometrically with C—H = 0.93 Å, and constrained to ride on their parent atoms, with *U*_iso_(H) = 1.2*U*_eq_(C).

## Synthesis and crystallization   

The title compound was prepared by the reaction of CoSO_4_·7H_2_O (1.40 g, 5 mmol) in H_2_O (25 ml) and pyrazine (0.40 g, 5 mmol) in H_2_O (25 ml) with sodium 4-formyl­benzoate (1.72 g, 10 mmol) in H_2_O (70 ml) at room temperature. The mixture was filtered and set aside to crystallize at ambient temperature for one week, giving orange single crystals.

## Supplementary Material

Crystal structure: contains datablock(s) I, global. DOI: 10.1107/S205698901500403X/wm5129sup1.cif

Structure factors: contains datablock(s) I. DOI: 10.1107/S205698901500403X/wm5129Isup2.hkl

CCDC reference: 1051344

Additional supporting information:  crystallographic information; 3D view; checkCIF report

## Figures and Tables

**Table 1:** Hydrogen-bond geometry (, ) *Cg*1 is the centroid of ring *A* (C2C7).

*D*H*A*	*D*H	H*A*	*D* *A*	*D*H*A*
O4H41O2	0.89(3)	1.72(3)	2.5909(16)	164(2)
O4H42O4^i^	0.71(3)	2.63(3)	2.958(2)	111(2)
C10H10O3^ii^	0.93	2.46	3.320(2)	154
C7H7*Cg*1^iii^	0.93	2.65	3.4216(15)	142

Symmetry codes: (i) 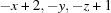; (ii) 
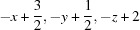
; (iii) 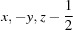.

**Table 2:** Experimental details

Crystal data	
Chemical formula	[Co(C_8_H_5_O_3_)_2_(C_4_H_4_N_2_)(H_2_O)_2_]
*M*_r_	473.29
Crystal system, space group	Monoclinic, *C*2/*c*
Temperature (K)	296
*a*, *b*, *c* ()	22.1623(6), 7.1193(2), 12.2911(3)
()	94.432(1)
*V* (^3^)	1933.49(9)
*Z*	4
Radiation type	Mo *K*
(mm^1^)	0.94
Crystal size (mm)	0.47 0.22 0.11

Data collection	
Diffractometer	Bruker SMART BREEZE CCD
Absorption correction	Multi-scan (*SADABS*; Bruker, 2012 ▸)
*T*_min_, *T*_max_	0.830, 0.914
No. of measured, independent and observed [*I* > 2(*I*)] reflections	27023, 2427, 2336
*R*_int_	0.024
(sin /)_max_ (^1^)	0.668

Refinement	
*R*[*F* ^2^ > 2(*F* ^2^)], *wR*(*F* ^2^), *S*	0.025, 0.071, 1.06
No. of reflections	2427
No. of parameters	154
No. of restraints	1
H-atom treatment	H atoms treated by a mixture of independent and constrained refinement
_max_, _min_ (e ^3^)	0.35, 0.34

Computer programs: *APEX2* and *SAINT* (Bruker, 2012 ▸), *SHELXS97* and *SHELXL97* (Sheldrick, 2008 ▸), *ORTEP-3* for Windows and *WinGX* (Farrugia, 2012 ▸) and *PLATON* (Spek, 2009 ▸).

## supplementary crystallographic information

### Crystal data

**Table:**

[Co(C_8_H_5_O_3_)_2_(C_4_H_4_N_2_)(H_2_O)_2_]	*F*(000) = 972
*M**_r_* = 473.29	*D*_x_ = 1.626 Mg m^−^^3^
Monoclinic, *C*2/*c*	Mo *K*α radiation, λ = 0.71073 Å
Hall symbol: -C 2yc	Cell parameters from 9866 reflections
*a* = 22.1623 (6) Å	θ = 2.4–28.3°
*b* = 7.1193 (2) Å	µ = 0.94 mm^−^^1^
*c* = 12.2911 (3) Å	*T* = 296 K
β = 94.432 (1)°	Block, orange
*V* = 1933.49 (9) Å^3^	0.47 × 0.22 × 0.11 mm
*Z* = 4	

### Data collection

**Table:**

Bruker SMART BREEZE CCD diffractometer	2427 independent reflections
Radiation source: fine-focus sealed tube	2336 reflections with *I* > 2σ(*I*)
Graphite monochromator	*R*_int_ = 0.024
φ and ω scans	θ_max_ = 28.4°, θ_min_ = 1.8°
Absorption correction: multi-scan (*SADABS*; Bruker, 2012)	*h* = −29→29
*T*_min_ = 0.830, *T*_max_ = 0.914	*k* = −9→9
27023 measured reflections	*l* = −16→16

### Refinement

**Table:**

Refinement on *F*^2^	Primary atom site location: structure-invariant direct methods
Least-squares matrix: full	Secondary atom site location: difference Fourier map
*R*[*F*^2^ > 2σ(*F*^2^)] = 0.025	Hydrogen site location: inferred from neighbouring sites
*wR*(*F*^2^) = 0.071	H atoms treated by a mixture of independent and constrained refinement
*S* = 1.06	*w* = 1/[σ^2^(*F*_o_^2^) + (0.0409*P*)^2^ + 1.5712*P*] where *P* = (*F*_o_^2^ + 2*F*_c_^2^)/3
2427 reflections	(Δ/σ)_max_ = 0.001
154 parameters	Δρ_max_ = 0.35 e Å^−^^3^
1 restraint	Δρ_min_ = −0.34 e Å^−^^3^

### Special details

**Table:**

Geometry. All esds (except the esd in the dihedral angle between two l.s. planes) are estimated using the full covariance matrix. The cell esds are taken into account individually in the estimation of esds in distances, angles and torsion angles; correlations between esds in cell parameters are only used when they are defined by crystal symmetry. An approximate (isotropic) treatment of cell esds is used for estimating esds involving l.s. planes.
Refinement. Refinement of F^2^ against ALL reflections. The weighted R-factor wR and goodness of fit S are based on F^2^, conventional R-factors R are based on F, with F set to zero for negative F^2^. The threshold expression of F^2^ > 2sigma(F^2^) is used only for calculating R-factors(gt) etc. and is not relevant to the choice of reflections for refinement. R-factors based on F^2^ are statistically about twice as large as those based on F, and R- factors based on ALL data will be even larger.

### Fractional atomic coordinates and isotropic or equivalent isotropic displacement parameters (Å^2^)

**Table:**

	*x*	*y*	*z*	*U*_iso_*/*U*_eq_	
Co1	1.0000	−0.05145 (3)	0.7500	0.01998 (9)	
O1	0.91572 (4)	−0.04830 (13)	0.80894 (9)	0.0276 (2)	
O2	0.86274 (5)	−0.17780 (18)	0.66579 (9)	0.0402 (3)	
O3	0.60114 (6)	−0.1312 (3)	0.95577 (12)	0.0651 (4)	
O4	0.96115 (5)	−0.06701 (18)	0.58454 (9)	0.0352 (2)	
H41	0.9245 (12)	−0.104 (4)	0.600 (2)	0.058 (6)*	
H42	0.9564 (12)	0.019 (4)	0.555 (2)	0.066 (8)*	
N1	1.0000	0.2518 (2)	0.7500	0.0247 (3)	
N2	1.0000	0.6436 (2)	0.7500	0.0235 (3)	
C1	0.86731 (5)	−0.11157 (17)	0.75983 (11)	0.0240 (2)	
C2	0.81095 (5)	−0.10250 (17)	0.82116 (10)	0.0226 (2)	
C3	0.81105 (6)	−0.0089 (2)	0.92052 (11)	0.0268 (2)	
H3	0.8467	0.0439	0.9518	0.032*	
C4	0.75794 (6)	0.0058 (2)	0.97289 (11)	0.0301 (3)	
H4	0.7580	0.0682	1.0394	0.036*	
C5	0.70463 (6)	−0.0726 (2)	0.92617 (12)	0.0292 (3)	
C6	0.70446 (6)	−0.1685 (2)	0.82755 (12)	0.0307 (3)	
H6	0.6689	−0.2222	0.7967	0.037*	
C7	0.75745 (6)	−0.18340 (19)	0.77557 (11)	0.0271 (3)	
H7	0.7574	−0.2478	0.7098	0.032*	
C8	0.64849 (8)	−0.0553 (3)	0.98296 (15)	0.0430 (4)	
H8	0.6472 (7)	0.029 (2)	1.0463 (12)	0.021 (4)*	
C9	0.97461 (6)	0.35053 (18)	0.82681 (11)	0.0287 (3)	
H9	0.9563	0.2869	0.8815	0.034*	
C10	0.97486 (7)	0.54530 (17)	0.82719 (12)	0.0282 (3)	
H10	0.9571	0.6090	0.8825	0.034*	

### Atomic displacement parameters (Å^2^)

**Table:**

	*U*^11^	*U*^22^	*U*^33^	*U*^12^	*U*^13^	*U*^23^
Co1	0.01683 (12)	0.01603 (12)	0.02763 (14)	0.000	0.00520 (8)	0.000
O1	0.0180 (4)	0.0288 (5)	0.0366 (5)	−0.0022 (3)	0.0058 (4)	−0.0040 (4)
O2	0.0284 (5)	0.0584 (7)	0.0351 (5)	−0.0116 (5)	0.0103 (4)	−0.0128 (5)
O3	0.0296 (6)	0.1092 (13)	0.0583 (8)	−0.0015 (7)	0.0149 (5)	0.0049 (9)
O4	0.0299 (5)	0.0449 (6)	0.0312 (5)	−0.0052 (5)	0.0051 (4)	0.0083 (5)
N1	0.0217 (7)	0.0178 (6)	0.0353 (8)	0.000	0.0065 (6)	0.000
N2	0.0242 (7)	0.0172 (6)	0.0301 (7)	0.000	0.0078 (6)	0.000
C1	0.0197 (5)	0.0204 (5)	0.0324 (6)	−0.0004 (4)	0.0059 (4)	0.0013 (5)
C2	0.0193 (5)	0.0218 (5)	0.0270 (6)	0.0006 (4)	0.0039 (4)	0.0022 (4)
C3	0.0224 (6)	0.0303 (6)	0.0272 (6)	0.0003 (5)	−0.0008 (5)	−0.0012 (5)
C4	0.0312 (7)	0.0337 (7)	0.0259 (6)	0.0040 (6)	0.0048 (5)	−0.0022 (5)
C5	0.0236 (6)	0.0332 (7)	0.0317 (6)	0.0046 (5)	0.0086 (5)	0.0055 (5)
C6	0.0207 (6)	0.0367 (7)	0.0351 (7)	−0.0048 (5)	0.0038 (5)	−0.0002 (6)
C7	0.0234 (6)	0.0308 (6)	0.0274 (6)	−0.0050 (5)	0.0048 (5)	−0.0036 (5)
C8	0.0316 (8)	0.0576 (11)	0.0417 (8)	0.0079 (7)	0.0152 (6)	0.0035 (7)
C9	0.0318 (6)	0.0211 (6)	0.0349 (7)	−0.0002 (5)	0.0137 (5)	0.0036 (5)
C10	0.0332 (7)	0.0210 (6)	0.0321 (7)	0.0016 (5)	0.0141 (5)	−0.0009 (5)

### Geometric parameters (Å, º)

**Table:**

Co1—O1	2.0551 (9)	C2—C1	1.5093 (17)
Co1—O1^i^	2.0551 (9)	C2—C3	1.3911 (18)
Co1—O4	2.1491 (11)	C2—C7	1.3961 (17)
Co1—O4^i^	2.1491 (11)	C3—H3	0.9300
Co1—N1	2.1588 (15)	C4—C3	1.3884 (18)
Co1—N2^ii^	2.1714 (15)	C4—H4	0.9300
O1—C1	1.2721 (16)	C5—C4	1.390 (2)
O2—C1	1.2451 (17)	C5—C6	1.391 (2)
O3—C8	1.205 (2)	C5—C8	1.478 (2)
O4—H41	0.89 (3)	C6—H6	0.9300
O4—H42	0.71 (3)	C7—C6	1.3836 (18)
N1—C9	1.3357 (15)	C7—H7	0.9300
N1—C9^i^	1.3357 (15)	C8—H8	0.984 (13)
N2—Co1^iii^	2.1714 (15)	C9—H9	0.9300
N2—C10	1.3347 (15)	C10—C9	1.3866 (19)
N2—C10^i^	1.3347 (15)	C10—H10	0.9300
			
O1—Co1—O1^i^	178.75 (5)	C3—C2—C1	120.92 (11)
O1—Co1—O4	91.46 (4)	C3—C2—C7	119.58 (12)
O1^i^—Co1—O4	88.60 (4)	C7—C2—C1	119.46 (11)
O1—Co1—O4^i^	88.60 (4)	C2—C3—H3	120.0
O1^i^—Co1—O4^i^	91.46 (4)	C4—C3—C2	119.94 (12)
O1—Co1—N1	89.38 (3)	C4—C3—H3	120.0
O1^i^—Co1—N1	89.38 (3)	C3—C4—C5	120.15 (13)
O1—Co1—N2^ii^	90.62 (3)	C3—C4—H4	119.9
O1^i^—Co1—N2^ii^	90.62 (3)	C5—C4—H4	119.9
O4—Co1—O4^i^	174.09 (7)	C4—C5—C6	120.13 (12)
O4—Co1—N1	92.96 (4)	C4—C5—C8	119.41 (14)
O4^i^—Co1—N1	92.96 (4)	C6—C5—C8	120.46 (14)
O4—Co1—N2^ii^	87.04 (4)	C5—C6—H6	120.2
O4^i^—Co1—N2^ii^	87.04 (4)	C7—C6—C5	119.67 (12)
N1—Co1—N2^ii^	180.000 (1)	C7—C6—H6	120.2
C1—O1—Co1	125.81 (9)	C2—C7—H7	119.7
Co1—O4—H41	96.6 (15)	C6—C7—C2	120.52 (12)
Co1—O4—H42	118 (2)	C6—C7—H7	119.7
H41—O4—H42	105 (3)	O3—C8—C5	125.34 (17)
C9—N1—Co1	121.75 (8)	O3—C8—H8	114.5 (10)
C9^i^—N1—Co1	121.75 (8)	C5—C8—H8	120.1 (10)
C9—N1—C9^i^	116.49 (15)	N1—C9—C10	121.79 (12)
C10—N2—Co1^iii^	121.61 (8)	N1—C9—H9	119.1
C10^i^—N2—Co1^iii^	121.61 (8)	C10—C9—H9	119.1
C10—N2—C10^i^	116.79 (15)	N2—C10—C9	121.57 (12)
O1—C1—C2	116.62 (11)	N2—C10—H10	119.2
O2—C1—O1	125.42 (12)	C9—C10—H10	119.2
O2—C1—C2	117.96 (11)		
			
O4—Co1—O1—C1	23.45 (11)	C3—C2—C1—O1	7.53 (18)
O4^i^—Co1—O1—C1	−150.64 (11)	C3—C2—C1—O2	−171.75 (13)
N1—Co1—O1—C1	116.39 (10)	C7—C2—C1—O1	−174.80 (12)
N2^ii^—Co1—O1—C1	−63.61 (10)	C7—C2—C1—O2	5.92 (18)
O1—Co1—N1—C9	35.39 (8)	C1—C2—C3—C4	176.79 (12)
O1^i^—Co1—N1—C9	−144.61 (8)	C7—C2—C3—C4	−0.9 (2)
O1—Co1—N1—C9^i^	−144.61 (8)	C1—C2—C7—C6	−176.64 (12)
O1^i^—Co1—N1—C9^i^	35.39 (8)	C3—C2—C7—C6	1.1 (2)
O4—Co1—N1—C9	126.82 (8)	C5—C4—C3—C2	−0.1 (2)
O4^i^—Co1—N1—C9	−53.18 (8)	C4—C5—C6—C7	−0.8 (2)
O4—Co1—N1—C9^i^	−53.18 (8)	C6—C5—C4—C3	1.0 (2)
O4^i^—Co1—N1—C9^i^	126.82 (8)	C8—C5—C4—C3	−179.86 (14)
Co1—O1—C1—O2	−3.6 (2)	C8—C5—C6—C7	−179.95 (14)
Co1—O1—C1—C2	177.23 (8)	C4—C5—C8—O3	−172.93 (18)
Co1—N1—C9—C10	179.66 (10)	C6—C5—C8—O3	6.3 (3)
C9^i^—N1—C9—C10	−0.34 (10)	C2—C7—C6—C5	−0.2 (2)
Co1^iii^—N2—C10—C9	179.66 (10)	N2—C10—C9—N1	0.7 (2)
C10^i^—N2—C10—C9	−0.34 (10)		

Symmetry codes: (i) −*x*+2, *y*, −*z*+3/2; (ii) *x*, *y*−1, *z*; (iii) *x*, *y*+1, *z*.

### Hydrogen-bond geometry (Å, º)

Cg1 is the centroid of ring *A* (C2–C7).

**Table:**

*D*—H···*A*	*D*—H	H···*A*	*D*···*A*	*D*—H···*A*
O4—H41···O2	0.89 (3)	1.72 (3)	2.5909 (16)	164 (2)
O4—H42···O4^iv^	0.71 (3)	2.63 (3)	2.958 (2)	111 (2)
C10—H10···O3^v^	0.93	2.46	3.320 (2)	154
C7—H7···*Cg*1^vi^	0.93	2.65	3.4216 (15)	142

Symmetry codes: (iv) −*x*+2, −*y*, −*z*+1; (v) −*x*+3/2, −*y*+1/2, −*z*+2; (vi) *x*, −*y*, *z*−1/2.
